# Training and mentorship as a tool for building African researchers’ capacity in knowledge translation

**DOI:** 10.1371/journal.pone.0266106

**Published:** 2022-03-31

**Authors:** Rose N. Oronje, Carol Mukiira, Elizabeth Kahurani, Violet Murunga

**Affiliations:** African Institute for Development Policy (AFIDEP), Nairobi, Kenya; Stellenbosch University, SOUTH AFRICA

## Abstract

As one of the main knowledge producers, researchers can play an important role in contributing to efforts that bridge the gap between knowledge, policy and practice. However, for researchers to play this role, they need knowledge translation (KT) capacities that many typically lack. Furthermore, research has confirmed that little is known on KT training approaches for LMICs researchers and their effectiveness. This paper seeks to contribute to filling this knowledge gap on KT training approaches for LMIC researchers by assessing the effectiveness of a training and mentorship intervention to build African researchers’ KT capacity. We conducted KT training and mentorship for 23 early and mid-career researchers from 20 universities in sub-Saharan Africa. This comprised a 5-day intense residential training workshop, followed by a 6-months mentorship. A pre- and post-training test was used to assess the immediate effect of the workshop. The intermediate effect of the training following a 6-month mentorship was assessed by the number of researchers who completed policy briefs during this period and those who participated in the webinar series conducted during this period. Overall, the aggregate average point change in the self-reported learning between the pre-training and the post-training survey was 1.9, which demonstrated the effectiveness of the training workshop. This was confirmed by a 33.7% increase in the aggregate average percentage of participants that responded correctly to questions assessing topics covered in the training between the pre-training and the post-training survey. During the mentorship period, 19 of the 23 researchers prepared and submitted complete drafts of their policy briefs within two months after the training. Fewer (4) researchers revised and submitted final policy briefs. More than half of the trained researchers participated in the webinars conducted in the first three months of the mentorship, whereas less than half of the researchers participated in the webinars conducted in the last three months. KT training and mentorship can be an effective intervention for addressing researchers’ KT capacity gaps. For sustainability, KT training and mentorship need to be integrated in graduate training programmes in universities so that future LMIC researchers leave training institutions with the KT capacities they need for influencing policy and programme decisions and actions.

## 1. Introduction

While research evidence is widely acknowledged as having an important role to play in improving development policy, programme and practice decisions [1–5], it often does not play this role for many reasons [6, 7]. This realisation has resulted in increased and sustained efforts to better understand effective ways through which research evidence could have a greater bearing on development policy and programme decisions and implementation. Researchers, who are among the main producers of research evidence, have an important role to play in promoting and enabling evidence use in development efforts. This is especially critical considering that among the main facilitators of research evidence use are: the availability of relevant and timely research, access to and improved dissemination of research, and a good understanding of the policy process and the context surrounding policy priorities by researchers [8, 9].

For researchers to play this important role in promoting and enabling evidence use, they need capacities that many typically lack [10]. Livny and others [11] noted that researchers’ weak or lack of “capacity to provide knowledge may frustrate attempts by potential ‘users’ to engage local actors in research and policy debate” (in [12], page 2). Lavis et al. [13] called on researchers to develop and sustain relationships with evidence users, collaborate with research users in the conduct of research, simplify and package research evidence in the language and formats that research users can understand, among others. In essence, researchers are now required to play an important role in promoting and supporting the use of evidence in policy, programme and practice decisions. A wide range of terminologies and definitions are used to refer to and define the process of promoting uptake of evidence into development policy, programme and practice decisions. This paper adopts the term Knowledge Translation (KT). The paper also adopts the Canada Institutes of Health Research (CIHR) definition of KT as *“a dynamic and iterative process that includes synthesis*, *dissemination*, *exchange and ethically sound application of knowledge to improve the health of people*, *provide more effective health services and products and strengthen the health care system”* [14].

Research has continued to confirm that researchers lack KT capacities. A recent review of published evidence on research translation capacity and practice by LMIC researchers by Murunga and others found that LMIC researchers “rarely practice KT, mainly because they face capacity constraints and barriers at individual and institutional levels” [10] (page 18). Among others, this study recommended the need for interventions that develop LMIC researchers’ KT knowledge and skills. This review also found limited quality research on LMIC researchers’ capacity and practice of knowledge translation, and called for more high quality and in-depth research to help address the KT capacity gap of LMIC researchers. This recent review confirmed the results of many other earlier studies on KT practice and capacity of LMIC researchers (see [15–20]).

KT training initiatives for researchers are still in their infancy [21, 22]. Mallido and others [21] review found that the few existing research on KT training was either descriptive or exploratory. A 2019 review by Tait and Williamson [22] found sparse published literature on KT training for health researchers. They also found the quality of the existing research to be low [22]. From the review, the focus of KT training for health researchers has been on KT theory, and developing practical skills in KT planning and evaluation, communication and relationship building [22]. Still from the review, the KT training interventions reviewed adopted a combination of different delivery styles for KT training for researchers as recommended by Mishra et al. [23]. These included instruction-type of delivery (mainly covering KT theory), and practise-based delivery to develop hands-on KT skills. Practise-based delivery included classroom practicals, mentorships, and secondments. Practise-based KT training delivery is recommended for ensuring effectiveness of the training intervention [24, 25].

Workshops, mentorships and secondments are reported as some of the common methods used in KT trainings [22]. Workshops have been demonstrated as effective methods in capacity strengthening, be it general research capacity or KT capacity [26–29]. Workshops have been said to enable hands-on training and learning, require active participant involvement, and facilitate exchange of information among participants [27, 30]. In three separate interventions in Nigeria, Uneke and others have demonstrated that workshops can be effective tools for building the capacity of researchers and policymakers in KT [12, 31]. Oronje et al. [32] also demonstrated the effectiveness of workshops in building the KT capacity for policymakers.

From the literature, mentorship is also emerging as a useful method in building KT capacity. Kho et al. [33] report on the use of brief mentorship sessions for KT trainees in small groups, which the trainees found to be useful in supporting their application of KT skills. More recently, Oronje et al. [32] used a 12-month mentorship programme to support the policymakers they trained in KT to apply the knowledge and skills learned. This involved provision of remote one-on-one support to trained policymakers by the trainers, and quarterly 1-day refresher workshops where the trainers facilitated discussions and sharing on KT topics identified by trainees as areas where they still needed more guidance. Oronje et al. [32] reported low participation in the mentorship sessions as well as slow progress in completion of KT products (by trained policymakers); in this intervention, about half of the trained policymakers participated in the mentorship programme, and a similar proportion completed their KT products through the mentorship programme.

The third and final method for KT capacity development for researchers emerging from the literature is secondments. Gerrish and Piercy [34] evaluated a secondment intervention to develop KT skills among researchers and clinicians, and found that the intervention increased KT knowledge and skills of the participants. Their intervention involved between 9–24 months of secondments. Although O’Donoughue and Anstey [35]’s assessment of a secondment programme for a researcher and a policymaker found it valuable in building KT capacity, they recommended the need to: test and assess different types of secondments, plan well for secondments with clear outputs and outcomes, and evaluate the effectiveness of secondments in building KT capacity. Uneke and others [29] tested a 6-month secondment intervention for researchers and policymakers, and found that the intervention increased KT knowledge and skills for each group.

In regard to evaluation of the KT training interventions, Tait and Williamson [22] report that there was minimal level of evaluation of the nine published interventions studied. In fact, only five interventions conducted any form of formal evaluation, whereas four relied on participant experiences. The five interventions that conducted some form of evaluation either used participant self-reported surveys and/or qualitative evaluation methods. In their paper, Uneke and others [31] note the use of self-reported pre-post surveys as one of the limitations of their study as participants may not be able to accurately assess their KT capacities. These issues notwithstanding, all the nine interventions reviewed by Tait and Williamson [22] reported that their interventions were well-received by training participants. Where the effects of the interventions were assessed, all studies reported that the interventions increased participants’ KT knowledge and skills [22].

From the above, it is clear that research on KT training for researchers is still a fledgling field. Therefore, many knowledge gaps still exist, including the relevant themes and effective approaches in delivering KT capacity programmes for researchers. The exploratory types of evaluations done on the small-scale interventions conducted to date mean that a lot more is needed to provide in-depth and proven guidance to future investments and programme development in this area.

To contribute to this emerging field of knowledge on KT training for researchers, and fill some of the noted knowledge gaps, this paper assesses the effectiveness of a training and mentorship intervention to build capacity of African researchers in KT (in this paper, this is termed as “research communication and policy engagement”). The paper draws on evidence from an intervention that sought to stimulate interest and develop the capacity of researchers from various universities in Africa in effective research communication and policy engagement. Specifically, the paper answers the question: How effective is training and mentorship as a tool for developing researchers’ capacities in KT?

## 2. Methods

The “*Research Communications and Policy Engagement”* training was part of the *Evidence Leaders in Africa (ELA)* project implemented for two years between November 2018 and November 2020. The ELA project was a partnership of the African Institute for Development Policy (AFIDEP) and the African Academy of Sciences (AAS). The main purpose of this partnership was to strengthen the capacity and leadership of distinguished researchers who are members of the AAS either as fellows or affiliates, or have received grants from the AAS (AAS grantees). The KT training programme was one of the interventions implemented under the ELA project, and it targeted AAS affiliates and grantees. AAS affiliates are young promising African scientists who have demonstrated prowess in the development and application of science in Africa [36]. At the time of the project, AAS had 87 affiliates. The focus of the training on AAS affiliates and grantees, and not AAS fellows, was deliberate since the training was targeting early and mid-career researchers, and so AAS fellows were not targeted as they are mostly senior researchers. The training aimed to equip early and mid-career researchers within the AAS with the knowledge and skills they need to promote and support the use of their research in government decision-making as they grow in their careers to become senior researchers.

### 2.1 Selection of researchers for the training programme

A call for applications for this training was circulated by AAS to its affiliates and grantees. This attracted 27 applicants out of which 26 were selected because they met the criteria outlined in the application. One applicant was neither an affiliate nor a grantee of the AAS and so was not selected for the training. Out of the 26 selected, 23 researchers turned up for the training workshop (from now on, these 23 researchers are referred to as “study participants”, “training participants”, or just “participants”). The 23 participants represented 9 African countries, and 20 universities in sub-Saharan Africa. 11 were women and 12 were men; all the 23 participants were PhD holders. The 23 participants were mainly involved in teaching and research at their universities (see Table 1). Majority of the participants (15 out of 23) were leading or involved in natural science research, 3 in multi-disciplinary research combining natural science and social science, 2 in social science research, and 1 in basic research. One participant did not provide a summary of their research.

**Table 1:** Roles of training participants at their universities.

Role	Number of training participants
1. Lecturer/Senior Lecturer & Research Fellow	12
2. Lecturer & Post-Doctoral Fellow	1
2. Researcher/Research Scientist	5
3. Post-Doctoral Fellow	4

*1 participant did not indicate their role at the university.

### 2.2 The KT training programme

The KT training programme had two components, namely: a one-week training workshop, and a follow-up mentorship programme (Table 2 presents the specific learning objectives of the different components of the training programme).

**Table 2:** Learning objectives of the KT training programme.

Element and Topics	Learning Objectives
***1*. *Training workshop***	• ***Enhance participants’ understanding of the*: *policymaking process*, *value and elements of strategic communication*, *elements of a policy brief*, *basics of visualising research data*, *and how to monitor and evaluate research influencing efforts***
	• ***Facilitate participants’ acquisition of hands-on skills in*: *analysing the policymaking process*, *developing a communications strategy*, *writing policy briefs*, *visualising research data*, *and monitoring and evaluation of research communication and policy engagement efforts***
1.1 Topic 1: Foundation of Policy-Making and Evidence Uptake	Participants will be able to:
	• Understand the context of policy-making
	• Understand the process of policy-making and the role of evidence
	• Identify barriers and facilitators of evidence use in decision-making
1.2 Topic 2: Strategic Communications	Participants will be able to:
	• Understand key elements of strategic communications
	• Prepare compelling messages for policy audiences based on research:
	○ Define a clear policy problem
	○ Present contextualised research findings relevant for addressing the policy problem defined
	○ Present clear implications of the research findings
	○ Present recommendations to address the policy problem
	• Prepare and present compelling policy presentations of research to a policy audience (drawing from preceding bullet)
1.3 Topic 3: Writing policy briefs	Participants will be able to:
	• Write clearly, concisely, and compellingly for policy influence
	• Understand policy briefs and their functions
	• Identify key elements and structure of a policy brief
	• Understand the content of each component of a policy brief
	• Critique policy briefs
	• Prepare a draft-0 of their policy brief
1.4 Topic 4: Visualising Research Data	Participants will be able to:
	• Understand the basics of visualising research data
	• Prepare visuals to communicate research
1.5 Topic 5: Monitoring and Evaluating Research Communications Efforts	Participants will be able to:
	• Understand the uniqueness in monitoring and evaluating research communications and policy engagement
	• Understand what to measure in research communication and policy engagement
***2*. *Mentorship programme***	• ***Support training participants in applying the skills acquired from the training workshop***
2.1 Mentorship on policy brief writing	• Participants will be able to complete the policy brief they started writing during the training workshop
2.2 Webinar series	Participants will gain understanding of:
	• A range of research communications topics not comprehensively covered during the training workshop
	• Additional research communications topics of interest not covered by the training workshop

#### 2.2.1 Training workshop

*Training content*. The training content was adapted from existing training modules developed and used by AFIDEP over the years. To customise the training to the needs of the 23 participants, we conducted a needs assessment two months before the training workshop, and used the results of this assessment to select specific modules to include in the training content. This was especially important given that research communication and translation, and engagement with policymakers entail a wide range of skills that cannot be covered adequately in a five-day workshop. The needs assessment therefore helped us to prioritise topics the 23 participants expressed most need and interest in building KT knowledge and skills. Table 3 below summarises the broad topics covered in the training workshop and the amount of time spent on each topic.

**Table 3:** Training topics and allocated time.

Topic	Time Allocation*
1. Foundation of policy-making and evidence uptake	3 hours
2. Strategic communications	7 hours
3. Writing policy briefs	7 hours
4. Visualising research data	3 hours
5. Monitoring and evaluating research communications efforts	1 hour

* Excludes the

• Time used for introductions on day-1 (to introduce participants, facilitators, training programme, and familiarization with training materials (2 hours), and the time used on day-5 to evaluate the effectiveness of the project (post-test) and outlining the mentorship programme (2 hours 15 minutes).

• Time/session on day-1 afternoon (3 hours) where participants presented their research to enable facilitators and peers understand their work. This was important as researchers were going to be using their research throughout the training in practical sessions (e.g. preparing effective presentations, writing policy briefs, etc.).

• Time participants practiced their policy presentations within small groups (i.e. working groups to receive feedback) (3 hours).

• Time participants used to give their final policy presentations to a “policy audience” (role played by facilitators) on the last day of the workshop (4 hours).

• Time participants spent preparing or revising their research presentations or writing their policy briefs during the training workshop week (this was often during breaks and evenings).

*Training delivery*. The training was delivered as an intensive, residential, five-day workshop on January 27–31, 2020 in Nairobi, Kenya. The training was facilitated by seven KT experts from AFIDEP and the AAS. The design and delivery of the training was underpinned by principles of adult learning and learner-centred pedagogy. The training was delivered using six interactive and participatory approaches including: Interactive lectures using PowerPoint presentations; Small group practical exercises (using case studies); Small group discussions; Individual practical exercises; Role plays; and Discussion panels of policymakers and researchers.

To facilitate provision of more personalised and relevant support to each individual participant, we divided the 23 participants into three workings groups prior to the workshop. Each working group had between 7–8 participants. Each working group was led by one of the facilitators who was a senior KT expert with extensive experience in research translation and policy engagement. The working group leader, was paired with another facilitator to support provision of individualised feedback to the 7–8 participants in the group. The three working groups were the channels through which participants presented their individual tasks done using their own research and got feedback from the group leader and members. The following practical exercises and tasks were reviewed and/or presented within the three working groups: Presentations of their research on day-1 and day-4 of the workshop; Review and feedback on policy presentations; and Review and feedback on policy brief outlines and draft-0 (prepared during the workshop), and later drafts of the policy briefs revised during the mentorship period.

#### 2.2.2 Six-months mentorship

While there is no universally agreed upon definition or form of mentorship [37–39], mentorship is seen as an “interactive, facilitative process meant to promote learning and development that is based on educational and social learning theories” [37] (page 2). For this study, mentorship took the form of a training follow-up and accompaniment activity to: (i) support training participants in their application of the knowledge and skills acquired from a training workshop, and (ii) provide an additional opportunity to address training topics not covered in-depth during the workshop, as well as, new KT topics of interest to participants, but which were not covered by the workshop. The first component on support for application of knowledge and skills acquired was focused on supporting participants to complete policy briefs that they started writing during the training workshop. The decision to focus on the policy brief as the main KT product during mentorship was informed by the fact that acquiring this skill was the main motivation of all participants for joining the training programme. The support on writing policy briefs was provided by trainers through one-on-one remote feedback to participants on their draft policy briefs. The second component of the mentorship programme was covered through a series of 1–2 hour webinars. The mentorship programme was implemented between February–July 2020.

*Mentorship on policy brief writing*. Through the training workshop session on “writing policy briefs”, participants started writing their policy briefs. By the end of the workshop, participants had prepared the draft-0 of their policy briefs and received feedback on these from their working group leaders. Participants were therefore required to revise and submit their draft-1 policy briefs within a month after the workshop. They were expected to complete their policy briefs by the end of the six-month mentorship period, working hand-in-hand with their working group leaders (i.e. mentors). The mentorship involved review and provision of feedback on draft policy briefs by the three working group leaders to individual participants via email. Specifically, one of the working group leaders adopted an active approach of writing each of her group members (participants) regularly (once every month) reminding them to submit their revised policy briefs. The other two working group leaders required the participants to drive the process, and did not therefore send regular reminders to their group members to submit their revised briefs.

*Webinar series*. We conducted five monthly 1–2 hour webinars. The webinar topics were identified through two ways. One way was by the training participants themselves (i.e. we used the post-training test to ask participants to indicate KT topics that they still wanted to learn more about). Using this method, 3 webinar topics were identified. The second way was by the training team–before the workshop, we knew that there were KT topics that we would either only have time to introduce at the workshop, and these would therefore require more time after the workshop to comprehensively cover, or would not introduce at all. This was the case for the *M&E of research communication and policy engagement efforts* topic, which we only introduced at the workshop, but covered in more depth in a webinar. The topic on media engagement was not introduced at the workshop at all due to lack of time, and so this was also covered through a webinar. The webinars targeted all the training workshop participants. Table 4 below lists the topics covered in the webinars and the number of participants who attended.

**Table 4:** Webinar topics and attendance.

Date	Topic	Number of Participants (n = 23)
March 17, 2020	Monitoring and evaluation of research communications and policy engagement	14
May 28, 2020	Social media for academics	13
June 12, 2020	Working with the media	11
July 1, 2020	Writing blogs and opinion pieces	4
July 15, 2020	Creating impactful infographics	7

### 2.3 Evaluating the training programme

#### 2.3.1 Evaluating the training workshop

*Pre- and post-training test*. To assess the immediate effect of the KT training workshop, we conducted a pre- and post-training test. The test comprised both questions that require participants to self-assess and report on their levels of knowledge and skills, as well as multiple-choice questions that test knowledge on issues covered to enable objective assessment. For the self-reporting assessment, participants were asked to rate either their understanding or level of knowledge of various KT components on a 5-point Likert scale, with 1 being the lowest and 5 the highest. Combining self-reported questions and knowledge questions strengthened our tool as it helped triangulate the participants’ self-reported perceptions with the responses to questions that test knowledge on issues covered for objective assessment. The pre-test was administered on the eve of the workshop, whereas the post-test was administered immediately after the last session of the workshop on day five; both surveys were administered via the Survey Monkey software (see the pre- and post-training test tool in S1 Appendix).

*Assessing participants’ skills in preparing policy presentations*. Among the key deliverables of the workshop was building the technical capacity of researchers to prepare and deliver impactful presentations of their research to policymakers, with clear “policy messages” (also referred to as “policy presentations” in this paper). Prior to the workshop, participants were required to prepare PowerPoint presentations of their research for policy audiences, and they made these presentations at the start of the workshop on day-1. Throughout the weeklong training, participants were required to revise their presentations to apply the effective communications knowledge and skills they were learning. The revised presentations were practised within the three working groups and feedback provided to individual participants by the working group leaders and fellow participants (on day-4 of the workshop). The final presentations were made on the final day of the workshop to “policy audiences” (role-played by trainers and participants). Besides using the pre- and post-test to capture participants’ views on the knowledge and skills acquired in regard to making effective policy presentations based on research, we also used a checklist to assess and provide feedback to participants on their policy presentations. The checklist, presented in S2 Appendix, was used by the trainers to provide feedback to participants during two working group sessions (on day-1 and day-4 of the workshop), and during the last-day session where participants presented their final policy presentations to “role-play” policy audiences. The checklist was not so much used as an evaluation tool for the policy presentations, but rather as a tool to capture and provide feedback that individual participants needed to improve their presentations. As such, we did not document systematic data on participants’ performance in each of the three sessions where trainers used the checklist. For the two working group sessions where participants made their policy presentations, they were allocated 10 minutes to present and 5 minutes to receive feedback mainly from the trainer and sometimes also from fellow participants. Therefore, the summary provided on how the participants’ policy presentations improved is based on overall views of the working group leaders (the trainers) based on the presentations made in the two working group sessions and the final-day plenary session.

#### 2.3.2 Evaluating the mentorship programme

The first component of the mentorship programme, which was supporting all 23 participants complete high quality policy briefs, was assessed through: (i) a review of policy brief drafts by working group leaders and provision of feedback covering: elements of a policy brief, content under each element of the policy brief, and the language (clear, free of jargon, and concise); and (ii) the number of participants who completed policy briefs. The second component, which was the webinar series, was assessed by the number of participants who attended each webinar.

Table 5 summarises what was evaluated for each of the two components of the KT training programme.

**Table 5:** What was evaluated in the training components.

Training components	What was evaluated in each training component
***1*. *Training workshop***	1. A pre- and post-training test comprising:
	• Questions that captured self-reported perceptions on the level of knowledge and skills acquired
	• Multiple-choice questions that test knowledge on issues covered to enable objective assessment
	2. For the element on preparing effective policy presentations, assessment was also done using a checklist (S2 Appendix). This checklist was used by the trainers during working group practice sessions, and during the last-day session where participants presented their final policy presentations.
***2*. *Mentorship***	
2.1 Writing policy briefs	1.Review of policy brief drafts by working group leaders and provision of feedback covering: elements of a policy brief, content under each element of the policy brief, and the language (clear, free of jargon, and concise).
	2.Levels of completion of policy briefs–how many participants completed their policy briefs?
2.2 Webinar series	1. Number of participants who attended each webinar.

### 2.4 Ethics statement

The African Institute for Development Policy (AFIDEP)’s ethics advisory committee waived the need for a prospective ethics review and approval for this study since the data used by the study is entirely numerical evaluative data that does not include any personally identifiable information. Researchers who participated in the training programme (i.e. study participants) upon which this study is based applied to be considered for the training the programme. Their applications for this training programme were in response to a Call for Applications that outlined all the components of the training programme, including the pre- and post-training workshop test that would be used to assess the effects of the training workshop. Participant data used for this study is based primarily on the results of the pre- and post-training workshop test. The preliminary section of the pre- and post-training workshop test tool informed participants that the data collected would be used to assess the effectiveness of the training programme to provide lessons for informing future training interventions in this area.

## 3. Results

### 3.1 Results of the pre-post training test

Tables 6 and 7 present results of the pre- and post-training test. Table 6 presents participants’ self-reported knowledge and skills, whereas Table 7 presents results of knowledge questions used to provide a more objective assessment of learning, and to provide data for triangulating the self-reported results. Overall, the average score in the self-reported learning changed from 2.2 before the training to 4.1 after the training, representing an aggregate average point change of 1.9 (see Table 6). This demonstrates the effectiveness of the training workshop. This is confirmed by a 33.7% increase in the aggregate average percentage of participants that responded correctly to questions assessing topics covered in the training between the pre-training and the post-training test (see Table 7). In the next sections, we discuss results of the specific learning components.

**Table 6:** Participants’ self-rating on KT knowledge and skills*.

Questions	Pre-test Average	Post-test Average	Point Change
1. Understanding of policymaking	2.0	3.7	1.7
2. Understanding of the stages of policymaking	1.8	4.2	2.4
3. Understanding of the role of evidence in policymaking	2.8	4.6	1.8
4. Understanding of the barriers and facilitators of evidence use in policymaking	2.2	4.4	2.2
5. Knowledge and skills in preparing effective policy presentations	2.0	4.2	2.2
6. Knowledge and skills in writing simply, clearly and compellingly or policy audiences	2.2	4.0	1.8
7. Knowledge and skills in writing policy briefs	2.1	3.8	1.7
8. Knowledge and skills in visualising data	2.9	4.1	1.2
**Aggregate Average**	**2.2**	**4.1**	**1.9**

* Participants were asked to rate their understanding and/or knowledge of the components in this table on a 5-point Likert scale, with 1 being the lowest and 5 the highest.

**Table 7:** Results of questions assessing knowledge acquired through training.

Questions	Percentage of participants who got the answer correct at pre-training stage (%)	Percentage of participants who got the answer correct at post-training stage (%)	Percentage Change (%)
1. Which of the following statements describe what a policy is?	11.7	27.2	15.5
2. Public policy can result from “non-decisions” (YES or NO)	41.7	100.0	58.3
3. The policymaking process is a …….. (Multiples provided)	41.7	63.6	21.9
4. Which of the following is NOT one of the stages of the policymaking process?	58.8	86.3	27.5
5. Which of the following are the three “streams” that need to align or merge in order to open a window of opportunity for policy influence?	0.0	31.8	31.8
6. Which of the following are key elements of a communications strategy?	70.5	100.0	29.5
7. Which of the following is NOT one of the strategies employed in creating a policy window of opportunity to influence policy decisions?	41.7	90.9	49.2
8. Which of the following are the two main categories of segmenting your research audiences?	0.0	95.4	95.4
9. What do you need to know about your audiences in order to communicate your research to them more effectively?	23.5	50.0	26.5
10. Which of the following is not a key element of a policy brief?	64.7	68.1	3.4
11. Effective monitoring and evaluation (M&E) for policy influence requires consideration of …….	47.0	59.0	12.0
**Aggregate Average**	**36.5**	**70.2**	**33.7**

#### 3.1.1 Knowledge of the policy-making process

Participants were asked to rate their understanding of various components of policy-making on a 5-point Likert scale with 1 being the lowest and 5 the highest (see Table 6, questions 1–4). Table 6 shows notable self-reported increase in knowledge of policy-making, with a point change ranging from 1.7 to 2.4. To triangulate the self-reported results, participants were asked five knowledge, multiple choice, questions on different aspects of the policy-making process (see Table 7, questions 1–5). Table 7 shows notable increases in knowledge on each of the four aspects of policy-making assessed. For question 1, it should be noted that, all multiple choices were correct and so participants needed to select the “all the above” choice. However, many participants did not select the “all the above” choice, but rather selected some of the other choices. This could explain why there was limited learning even after the training. Questions 3–5 focused on theory in policy-making, and even though there was increase in knowledge recorded, some notable percentage of participants achieved little learning. Question 3, for instance, was about the complexity, messiness and non-linearity of the policy-making process, but as seen in Table 7, 36.4% of participants still did not have a good understanding of this even at the end of the workshop. On question 4 on the stages of the policy-making process, 13% of participants still did not understand this fully by the end of the workshop. Question 5 was perhaps the most difficult as it focused on a specific theory, i.e. John Kingdon’s [40] three streams theory of how issues rise on the political agenda. All participants had no knowledge of this theory before the workshop, and even after the workshop, only 31.8% fully understood the theory. Nevertheless, the results of both the self-reported and the knowledge questions show that the training increased participants’ knowledge and understanding of the policy-making process.

#### 3.1.2 Strategic communications knowledge and skills

Participants were assessed on their knowledge and skills relating to various aspects of strategic communications as shown in Tables 6 and 7 (questions 5 and 6 in Table 6, and questions 7, 8 and 9 in Table 7). Question 5 in Table 6 assessed participants’ skills on preparing effective policy presentations, and shows a 2.2 point change in skills between pre-training to post-training. This result is triangulated by the trainers’ overall assessment of the final policy presentations made by participants on the final day of the workshop, which showed that all participants presented completely transformed presentations at the end of the workshop. Specifically, most participants made presentations that: had notably improved content including definition of a clear policy problem, presented only the research findings that address the defined policy problem, clear implications of the research findings, and recommendations that flow logically from the implications of the research findings and address the policy problem defined; used simple, concise and clear language free of jargon; and used visuals to communicate research. Furthermore, participants used their presentation time (12 minutes) more strategically, ensuring that they used their first few minutes of their presentation to capture the attention of policymakers by making a compelling case of why policymakers needed to address the policy problem urgently, and then using most of the presentation time to present research findings and their implications for the policy problem, and the actions needed to address the policy problem (recommendations).

The training also had sessions where participants learned about principles of “writing simply” for influence, and had opportunities to practice individually and in small groups to rewrite complex research texts in simple, concise and compelling ways for policy audiences. Question 6 in Table 6 assessed participants’ skills on this and shows a 1.8 point change in skills between pre-training and post-training.

Acquiring skills in writing policy briefs was one of the areas where all training participants had expressed a great need. The training workshop had sessions where participants learned about policy briefs, their purpose and function, and their structure/key components. Each participant had an opportunity to start writing a policy brief based on their issue of interest and using their own research or research from elsewhere. Question 7 in Table 6 assessed participants’ skills on this and shows a 1.7 point change between pre-training and post-training. In Table 7, we also assessed knowledge on policy briefs using question 10, but it appears participants started with high-levels of knowledge on the components of a policy brief at 64.7% at pre-training. However, there was little learning as only 68.1% of participants got this response correctly after the training. Given that the question was quite basic (key elements of a policy brief) and was covered extensively in the workshop (through an interactive lecture session, and small group discussions that critiqued sample policy briefs using a checklist of key elements of policy briefs and presented back in plenary), the little learning recorded is hard to explain.

Visualising research data in order to make it more accessible and easy to understand by policy audiences was one of the areas of interest where participants wanted to acquire skills. Question 8 in Table 6 assessed participants’ skills on this and shows a 1.2 point change in skills between pre-training and post-training.

Another important aspect of the training was developing researchers’ knowledge and skills in developing and implementing communications and policy engagement strategies. Question 6 in Table 7 assessed knowledge and skills on this and shows that the training was most successful here with 100% of participants being able to identify all elements of a communications strategy. The training sessions on communications strategy further introduced participants to effective strategies in creating windows of opportunity for policy influence. This particular session drew partly from the Kingdon’s theory [40] to focus on effective strategies for merging or aligning the three streams in order to create a window of opportunity for change. Question 7 in Table 7 assessed participants’ knowledge on these strategies, and shows a 49.2 percentage change in knowledge, with 90.9% of the participants answering the question correctly post-training. This result is interesting considering that participants struggled to understand Kingdon’s theory (as reported earlier) [40], but this result shows that most participants understood how to apply the theory in their efforts to open windows of opportunity for policy influence.

Finally, questions 8 and 9 in Table 7 assessed participants’ understanding of the target audiences for their strategic communications efforts. This was a critical element of the training workshop given the central importance of understanding policy stakeholders, and developing and sustaining meaningful relationships, in effective KT efforts. Results of question 8 show that participants recorded notable understanding of the broader stakeholder categories for research evidence. However, question 9 shows that quite average learning was realised on stakeholder analysis, yet this is critical for designing effective stakeholder engagement/communication strategies. The limited learning on stakeholder analysis could be explained by the fact that the question required participants to choose all the correct responses among listed multiple choices (six multiple choices were provided of which four were correct). At the end of the workshop, half of the participants were able to identify all the correct responses among the multiple choices, while half were not. Therefore, this does not mean that half of the participants did not learn anything, but that they were unable to identify all the correct responses to the question.

#### 3.1.3 Monitoring and evaluation of research communication and policy engagement efforts

Developing researchers’ understanding of how to measure success of their KT efforts was an important component of the training. However, due to limited time, this topic was only introduced at the workshop through a one-hour session, and then covered in a bit more detail through a webinar conducted after the workshop. Question 11 in Table 7 assessed knowledge on this topic and shows only a 12 percentage change between pre-training and post-training. This low level of learning could be explained by the fact that this topic was only introduced at the workshop, but not covered exhaustively as had been planned before the workshop because we ran out of time. While the topic was later revisited through a webinar during the post-training mentorship period to cover the content not covered at the workshop, the post-test result reported here was captured at the end of the training workshop.

### 3.2 Results of the post-training mentorship

#### 3.2.1 Mentorship on policy brief writing

During the six-month mentorship, researchers were required to complete their policy briefs and submit these to their working group leaders for review and feedback. Out of the 23 participants, 19 submitted a first complete draft (1^st^ draft) of their policy brief in 1–2 months after the training workshop. Feedback was sent to all the 19 researchers on the first draft of their policy briefs within 1 month. Out of the 19, nine researchers revised their policy briefs and resubmitted revised briefs (2^nd^ draft). Feedback was sent to the nine researchers. At this stage, most of the feedback indicated the final revisions that needed to be made for them to then resubmit their final briefs. Out of the nine, only four submitted their final policy briefs. The low turnaround at the second stage of the policy brief writing process could be partly explained by the differences in strategy of the working group leaders in supporting researchers complete their policy briefs, where one of the leaders wrote her group members regularly reminding them to submit their revised policy briefs, while the other two leaders did not. As a result, among the nine researchers who submitted their revised/second draft policy briefs, seven belonged to the group coordinated by the leader who sent regular reminders to her team members to submit their revised briefs (see Table 8). However, this strategy appears to have had little effect on the submission of final policy briefs as only two participants who received regular reminders submitted final briefs, the same number as those who did not receive regular reminders.

**Table 8:** Policy brief writing and completion outcomes.

	Completed and submitted Draft-1 Policy Briefs	Revised and submitted revised Draft-2 Policy Briefs	Revised and submitted Final Policy Briefs
Working Group 1 comprising 9 participants	8	7	2
Working group 2 comprising 7 participants	5	0	0
Working Group 3 comprising 7 participants	6	2	2
**Total **	**19 **	** 9**	**4**

#### 3.2.2 Webinar series

As noted earlier, we conducted five webinars (each lasting between 1–2 hours) as part of the six-months mentorship programme. The attendance for each of the webinars by trained participants ranged from four to 14 out of the 23 participants (see Table 4 (in Methods section)). This represents very low to average levels of participation in the webinars. The first two webinars were most attended, i.e. on M&E of research translation, and Social Media for academics, each attracting more than half of the trained researchers (14 and 13 participants, respectively). The webinar on “Working with Media” was third in terms of attendance, attracting 11 participants. The webinar on writing blogs and opinion pieces was the least attended, attracting just 4 participants out of the 23 participants. This was followed by the webinar on “Creating impactful infographics”, which attracted 7 participants. Table 9 summarises webinar attendance. None of the 23 participants attended all the five webinars. Only 3 participants attended four out of the five webinars, 5 participants attended three webinars, 10 participants attended two webinars, and 5 participants attended only one webinar. One participant did not attend any of the five webinars.

**Table 9:** Webinar attendance summary.

Number of webinars	Number of Attendees (n = 23)
All 5 webinars	0
4 out of 5 webinars	3
3 out of 5 webinars	5
2 out of 5 webinars	10
1 out of 5 webinars	5
0 out of 5 webinars	1

Based on the data, the first two webinars conducted within four months after the training workshop attracted more than half of the researchers compared to the last three webinars conducted in the fifth and sixth months after the workshop. While participants’ attendance of webinars was likely influenced by many factors, including interest in the webinar topic and availability for the webinar slot, there could be some influence by the timing, i.e. as the months went by after the training workshop, researchers lost the momentum gained from the workshop, and therefore only few found time for the webinars. We noted a similar trend with the policy briefs completion, with more researchers being able to submit revised policy briefs within the first 2–4 months, than within the last 5–6 months of the mentorship period.

## 4. Discussion

This study sought to contribute to the emerging field of knowledge on KT training for researchers by assessing the effectiveness of a training and mentorship intervention to build capacity of African researchers in knowledge translation. The results of the pre- and post-training test, and mentorship programme demonstrate the effectiveness of the training and mentorship programme. These results are in line with the results of other KT training interventions conducted in the last couple of years [12, 31, 32]. In view of the notably weak capacity of researchers in KT, and the lacking KT training programmes in universities [10], the demonstrated effectiveness of this training and mentorship programme mean that it is an important intervention for future efforts. Indeed, the programme could be offered as a short course to graduate students in universities or ideally, integrated into graduate and post graduate degree training curricula, in efforts to address the huge capacity gap in KT among LMIC researchers [10]. This would ensure that the future crop of LMIC researchers would leave training institutions with the KT capacity they need to influence policy and programme decisions and actions. The KT training and mentorship programme is also a valuable intervention for “practicing” LMIC researchers as demonstrated by this study and should be tested and adapted by other scholars in diverse settings including LMIC academic institutions.

As noted by Tait and Williamson [22], past KT training programmes have had minimal levels of evaluation. Studies have either used participant self-reported surveys to assess the knowledge and skills gained, or only captured participant experiences at the end of the trainings. This study improved on the evaluation of the effectiveness of the training programme in two ways. First, the study included knowledge questions in the tool used for the pre- and post-training tests. The inclusion of these types of questions provided results that triangulated the results of the self-reported questions. Secondly, we used two substantive practical exercises to enable application of the knowledge and skills acquired from the training as an intermediate outcome of the training. One was the presentations that participants made at the start of the workshop and then revised and presented at the end of the workshop, based on learning from the workshop. We reported notable transformation of the presentations that demonstrated the learning realised during the workshop. Second was the requirement for participants to write policy briefs as the main deliverable of the mentorship period. While all the researchers came to the workshop with no capacity in writing policy briefs, 19 of the trained researchers had prepared policy briefs by the end of the mentorship period. The results of these two practical deliverables, requiring participants to apply the knowledge and skills acquired in the training strengthened the effectiveness of the training.

Like past KT trainings for researchers, our training covered KT theory, planning and strategies informed by KT theory, and developing practical skills in research communications and relationship building. From our results, researchers recorded more learning in practical skills in translating their research into accessible language and products (preparing and delivering effective policy presentations, writing for policy influence, writing policy briefs), than knowledge in KT theory, stakeholder analysis, and strategies for creating windows of policy influence. This may be because of two reasons. The first is that often more time is needed for learners to develop in-depth understanding of new theories and applying the theories in designing effective strategies. Therefore, the time allocated to the sessions on KT theory and strategies may have been inadequate, and consequently minimal learning was recorded. The second is that the primary reason for joining the training for majority of the researchers was to acquire practical skills in translating their research into non-scientific evidence products (such as policy briefs). As such, the researchers were likely more keen to complete the training programme with the practical skills they needed than the theoretical knowledge on KT. Effective KT efforts need capacity in both KT theory and strategies, as well as practical skills in translating research into accessible messages and formats. Therefore, future KT training programmes need to find a balance in effectively building knowledge and skills in theory and strategies, as well as, in practical skills in translating complex research into accessible messages and formats for non-technical audiences.

Post-training mentorship has been noted as important in helping learners apply the knowledge and skills acquired from training programmes [32]. In this study, the post-training mentorship programme supported researchers in completing their policy briefs, as well as covering topics that the 5-day workshop was not able to cover. As such, the results of this study confirm the usefulness of post-training mentorship programmes in supporting learners to apply knowledge and skills acquired. Importantly though, this study shows that post-training mentorship programmes may be more effective if implemented immediately after the workshop for a few weeks or months, than if implemented over a longer period of time after the workshop. Our results showed that the webinars conducted within four months after the workshop attracted better attendance than those conducted in the fifth and sixth months after the workshop. Also, while 19 out of 23 researchers submitted the first complete draft of their policy briefs within 2 months after the training workshop, fewer researchers effected the revisions recommended and resubmitted revised policy briefs in later months as was required. More research is, however, needed to confirm this. On the numbers of researchers who revised and completed their policy briefs during the mentorship period, there could also be some influence of the approaches used by the three mentors. The results suggest that an active follow-up approach by mentors could generate better results than expecting researchers to drive the process of review and completion of their KT products, but more research is needed to confirm this as this difference was only observed at the second stage of submitting revised policy briefs and not at the last stage of submitting final policy briefs.

This, however, brings to light the need to identify incentives for researchers to complete their evidence products. For instance, offering to publish the final policy briefs (or whatever KT products researchers work on during mentorship such as blogs, infographics, etc.) could be an incentive. This is because the few researchers who submitted final policy briefs were disappointed that we were not able to publish their final policy briefs, and requested us for advice on where to publish their briefs. Beyond publishing researchers’ KT products, researchers could be trained and encouraged to view their KT activities as an academic exercise by systematically documenting them and tracking their effects/impacts, results of which they can publish in scientific journals to contribute to the evidence base on KT.

It is important to note the important role the motivation and interest of the researchers played in enabling the learning realised. All the researchers applied to join the training, and as part of the application, they were required to indicate why they needed the training. If this KT course is offered to researchers who have little or no interest in KT, it may have different results.

Finally, it is important to note that individual capacity development interventions like the one presented in this paper need to go hand-in-hand with institutional capacity interventions [10, 32, 41]. Other studies have shown that many research institutions in LMICs lack or have weak institutional capacity (including structures, tools, processes and systems) to promote and enable researchers’ practice of KT [10] (page 18).

The study has four limitations. One is the point noted earlier relating to the trained researchers being already interested in KT because they applied for the training programme, which means that the results of the training programme are not generalisable to other researchers who may not have interest in KT. The second is that we did not conduct a comprehensive evaluation of the mentorship programme. Besides capturing the number of revised and completed policy briefs, and the number of participants who attended each of the webinars hosted, we did not capture other evaluative data on the programme. The third is that we did not assess participants’ reactions or experiences with the training programme (e.g. through interviews with participants). This could have helped explain the results of the training workshop and mentorship programme as well as the challenges faced. Further, the data arising from this could have provided additional insights relevant for improving future KT training programmes. The fourth limitation is that the authors of this paper were involved in the design and delivery of the training programme, and therefore, study results may be subject to self-reporting bias. However, it is important to note that the results of this study on the effects of KT training do not contradict results of earlier studies. Also, given the limited knowledge on effective KT training approaches for researchers, the study offers useful insights needed for informing future efforts.

## 5. Conclusions

Researchers can play an important role in contributing to bridging the gap between knowledge, policy and practice. For researchers to play this role, however, they need KT capacities. This paper has contributed to the emergent knowledge on KT capacity development for LMIC researchers. The paper not only demonstrates that KT training and mentorship can be an effective intervention for addressing researchers’ KT capacity gaps, but also generates lessons to improve future design and implementation of similar interventions. For sustainability, KT training and mentorship need to be integrated in graduate training programmes in universities so that future LMIC researchers will leave training institutions with the KT capacities they need for policy influence.

## Supporting information

S1 Appendix Pre- and post-training test tool. (PDF)

S2 Appendix Checklist used for assessing policy presentations. (PDF)
