# Supplementary material for: Training and mentorship as a tool for building African researchers’ capacity in knowledge translation
Source: PLoS One. 2022 Mar 31;17(3):e0266106. doi: 10.1371/journal.pone.0266106 (PMC8970368; doi:10.1371/journal.pone.0266106)
Supplement: S1 Appendix — (PDF) [file pone.0266106.s001.pdf]

S1 Appendix: Pre- and Post-Training Test Tool

A. Self-reporting questions

Rate your understanding of the following, with 1 being low and 5 high:

	Rating				
Concepts	***1***	***2***	***3***	***4***	***5***
1. Policymaking					
2. The stages of the policymaking process					
3. The role of evidence in the policy-making					
4. Barriers and facilitators of evidence use in policymaking processes					

5. Rate your knowledge and skills in preparing effective policy presentations, with 1 being poor and 5 being excellent.

i. 1
ii. 2
iii. 3
iv. 4
v. 5

6. Rate your knowledge and skills in writing simply, clearly, and compellingly for policy audiences, with 1 being poor and 5 being excellent.

i. 1
ii. 2
iii. 3
iv. 4
v. 5

7. Rate your knowledge and skills in writing policy briefs, with 1 being poor and 5 being excellent.

vi. 1
vii. 2
viii. 3
ix. 4
x. 5

8. Rate your knowledge and skills in visualizing data, with 1 being poor and 5 being excellent.

i. 1
ii. 2
iii. 3
iv. 4
v. 5

B. Knowledge-testing questions

1. Which of the following statements describe what a policy is?

i. A course or principle of action adopted or proposed by a government, party, business, or individual
ii. Decisions, plans, and actions that are undertaken to achieve specific health care goals within a society
iii. When tackling an issue is simply avoided
iv. (i) and (ii)

v. All of the above

2. Public policy can result from “non-decisions”.
i. True
ii. False

3. The policymaking process is a:
i. Complex, messy, power-laden, and interest-driven
ii. Linear, moving from agenda setting, to policy formulation, to implementation, to evaluation
iii. All of the above

4. Which of the following is NOT one of the stages of the policymaking process?
i. Implementation
ii. Agenda setting
iii. Formulation
iv. Research generation
v. Evaluation

5. Which of the following are the three “streams” that need to align or merge in order to open a window of opportunity for policy influence?
i. Political environment
ii. Problems
iii. Research evidence
iv. Solutions

6. Which of the following are key elements of a communications strategy?
i. Goals
ii. Objectives
iii. Messages
iv. Evaluation
v. All of the above

7. Which of the following is NOT one of the strategies employed in creating a policy window of opportunity to influence policy decisions?
i. Policy learning
ii. Publishing research
iii. Attention focusing
iv. Policy community strengthening

8. Which of the following are the two main categories of segmenting your research audiences?
i. Policy actors
ii. Primary audiences
iii. Media audiences
iv. Advocacy stakeholders
v. Secondary audiences

9. What do you need to know about your audiences in order to communicate your research to them more effectively?
i. Their knowledge of your research issue
ii. Their sources of information

iii. Their political interests
iv. The number of their constituents
v. Their level of influence
vi. All the above

10. Which of the following is not a key element of a policy brief?
i. Problem and policy-oriented
ii. Analysis-driven
iii. Evidence-based
iv. Literature review
v. Offers viable recommendations
vi. Appealing layout

11. Effective monitoring and evaluation (M&E) for policy influence requires consideration of:
i. Shifting contexts
ii. Relationships and actors
iii. Different sources of evidence
iv. Unexpected effects
v. (i) and (ii)
vi. All of the above
