# Supplementary material for: Training and mentorship as a tool for building African researchers’ capacity in knowledge translation
Source: PLoS One. 2022 Mar 31;17(3):e0266106. doi: 10.1371/journal.pone.0266106 (PMC8970368; doi:10.1371/journal.pone.0266106)
Supplement: S2 Appendix — (PDF) [file pone.0266106.s002.pdf]

S3 Appendix: Checklist used to assess and provide feedback to participants on their policy presentations

Element	Yes/No	Comments
1. Content of presentation		
1.1 Is the policy problem clearly and concisely defined?		
1.2 Is the policy problem contextualised to make it salient? (why should a policymaker pay attention to this problem?)		
1.3 Do the research findings presented address the policy problem defined?		
1.4 Are the research findings presented in a clear and concise manner?		
1.5 Are implications of the research findings presented to help the policymaker appreciate why the research results are important?		
1.6 Are recommendations presented emanating from the research findings and the implications presented?		
1.7 Are the recommendations specific?		
2. Language		
2.1 Was the presentation free of research/technical jargon?		
2.2 Was language used clear and compelling?		
3. Use of visuals: Were appropriate and easy to understand visuals used in the presentation?		
4. Was the presentation made in an engaging manner? (presenter's confidence, eye contact audience, etc.)		
5. Time management		
5.1 Was presentation made within the allocated 12 minutes?		
5.2 Did the researcher use the time allocated appropriately? (12 minutes were shared adequately to describe the policy problem; present the research findings and explain their implications; and present recommendations)		
